# Congenital Diaphragmatic Hernia with Delayed Presentation

**DOI:** 10.1155/2016/7284914

**Published:** 2016-10-30

**Authors:** Alireza Malekzadegan, Alireza Sargazi

**Affiliations:** ^1^Department of Thoracic Surgery, Zabol University of Medical Sciences, Zabol, Iran; ^2^Student Research Committee, Zabol University of Medical Sciences, Zabol, Iran

## Abstract

Congenital diaphragmatic hernia (CDH) is caused due to abnormal formation of the muscular parts of diaphragm. The incidence of CDH in common births ranges from 1/25000 to 1/30000. Pulmonary hypoplasia and pulmonary hypertension are factors that associate with the increase of mortality and morbidity due to CDH. We presented a 68-year-old Iranian woman with abdominal pain and tenderness in right upper quadrant who was diagnosed as having CDH. The disease was detected using chest X-ray and chest and abdomen sonography and confirmed with chest and abdomen CT scan with and without oral contrast. A defect was revealed in posterolateral right diaphragm with omentum and transverse colon herniated through it. Right posterolateral thoracotomy was performed to cure the disease. CT and CXR were the two useful methods in diagnosis of CDH in this patient, although CDH detection prior to surgery is too challenging because of rare cases and different types of CDH. In order to improve clinical cares in adult CDH patients, investigating more cases and long term follow-up are recommended.

## 1. Introduction

Congenital diaphragmatic hernia (CDH) occurs due to incomplete muscularization of the diaphragm. The rate of CDH ranges from 1 : 2500 to 1 : 3000 live births, and pulmonary hypoplasia and pulmonary hypertension are associated with the increase of mortality and diseases [1]. In 15–20% of cases with CDH, the hernia occurs on the right side and in 80–85% of subjects on the left side. The diaphragm is rarely engaged in both sides [1, 2]. The survival of these patients is estimated to be 55–65% [3].

CDH includes Bochdalek hernia (70%) in the posterior-lateral and Morgagni hernia (25–35%) in the anterior or central (2–5%) part of the diaphragm [4]. Despite the high prevalence of Bochdalek hernia during infancy, the disease is rare in adults and the diagnosis of this type of hernia is very difficult and, in most patients, it is not diagnosed due to the mild delayed manifestation of CDH. Patients with delayed manifestation of CDH have better prognosis than patient with early manifestation. Small intestine finds a way into thoracic hernia more than any other abdominal organs [5]. The most common clinical manifestation in infants is respiratory distress while, in adults, mild respiratory and gastrointestinal symptoms are more prevalent, and 25% of the hernia is asymptomatic [6]. Respiratory symptoms are prominent in the right hernia while left hernia shows itself by gastrointestinal symptoms. Moreover, the short-term pulmonary results of patients with right CDH are not worse than those of patients with left CDH [1]. Clinical presentation of CDH in adults is summarized in Table 1 [7, 8]. In this case study, we have reported an elderly patient with right congenital diaphragmatic hernia diagnosis and delayed gastrointestinal clinical manifestation.

## 2. Case Presentation

**Figure 1:**
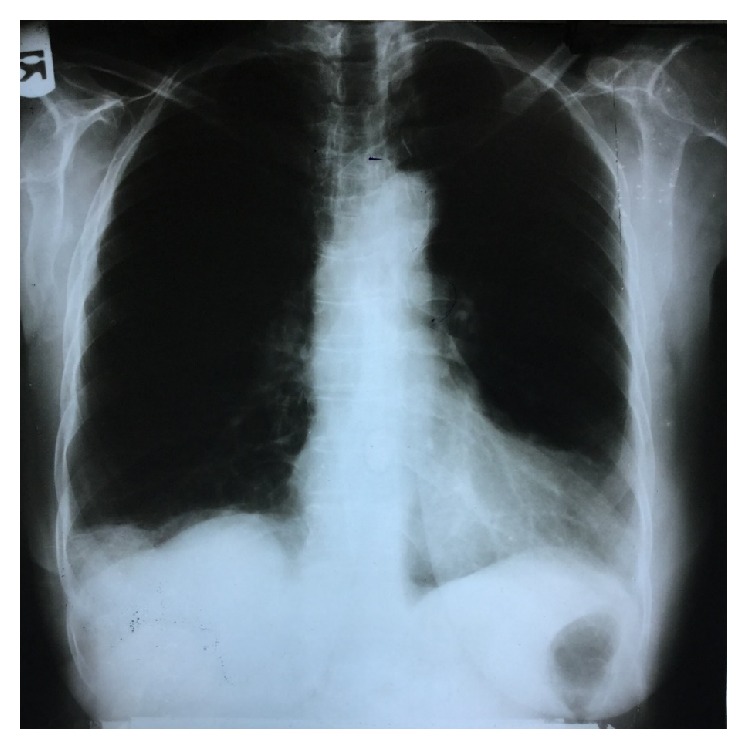
X-ray chest showing right diaphragmatic hernia.

**Figure 2:**
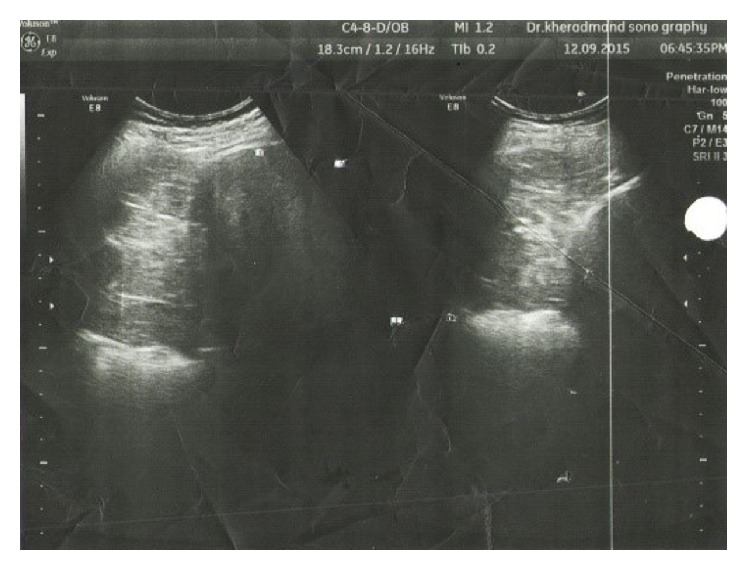
Sonography chest.

**Figure 3:**
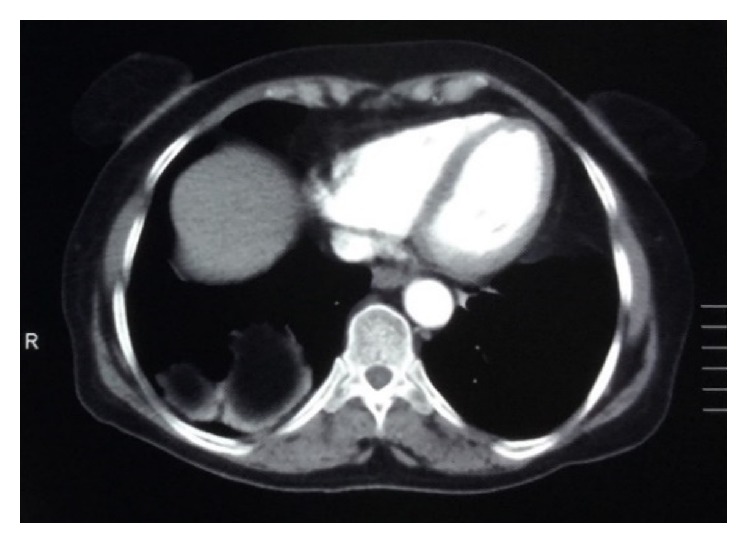
Contrast-enhanced CT scan showed right diaphragmatic hernia.

**Figure 4:**
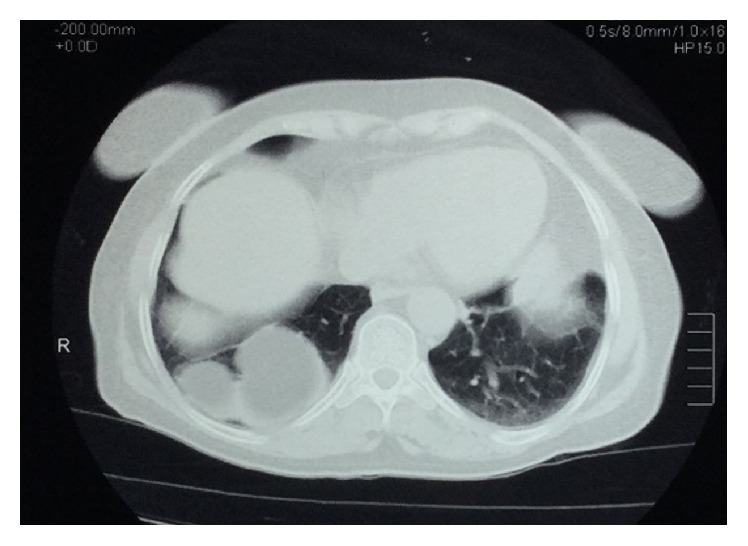
Contrast-enhanced CT scan showed right diaphragmatic hernia.

The patient is a 68-year-old woman with a 2-day history of constant and sharp pain on the right side of abdomen who has come to emergency department. The pain increases by eating and decreases by lying down. Her bowel habits are normal. There are no respiratory signs and her cardiorespiratory examination is normal. Her abdomen examination shows that there is tenderness in the right upper quadrant (RUQ) below the costal margin. Chest examination is normal and chest radiograph shows hyperlucency of the right lower lobe of lung (Figure 1). Abdomen and pelvic ultrasound were requested for further examination and a solid mass with the size of 94 × 67 mm was observed in the right lower lobe of the lung (Figure 2). Then, in order to detect the location of lesion precisely, the axial cross-sectional chest CT scan was done with and without intravenous oral contrast and a mass was observed in the posterior side of the right diaphragm with omentum herniated in it (Figures 3 and 4). The patient with diagnosis of the right diaphragmatic hernia was moved to thoracic surgery unit.

**Figure 5:**
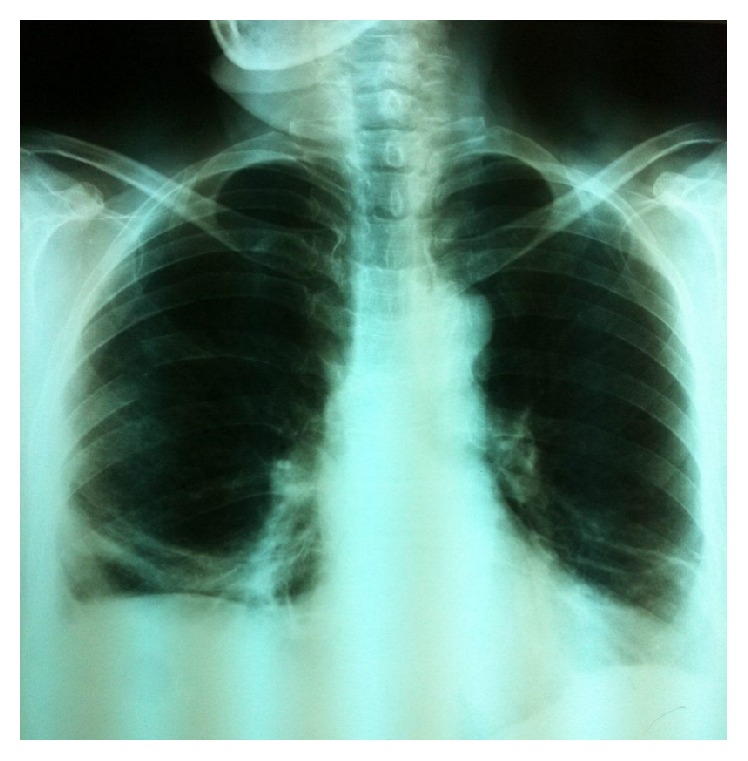
X-ray chest after surgery (thoracotomy).

The patient has undergone posterior-lateral right sided thoracotomy to repair the defect. Transverse colon and omentum were herniated to the chest at costophrenic angle. The size of diaphragm defect was about 4 × 6 cm^2^. Hernia sac was separated from diaphragm and the diaphragm was opened and the contents were directed into the abdomen. Then the diaphragm was repaired and covered with prolene mesh. After 24-hour care at ICU, the patient was transferred to the surgical ward.  Figure 5 shows the patient's chest X-ray after the surgery. The patient got full recovery and was discharged from the hospital.

## 3. Discussion

Bochdalek hernia was described by Bochdalek for the first time in 1834 with congenital defect of posterior-lateral part of diaphragm without hernia sac. Due to the complexity of congenital diaphragmatic hernia the factors involved in the development of Bochdalek hernia are unknown; however, at present its cause is expressed to be the lack of closure of pleural and peritoneal cavity due to disruption of molecular signaling during organogenesis during the 9th to 10th weeks of pregnancy [9–11]. Bochdalek hernia is associated with chromosomal disorders (10–25%) and other congenital defects (25–57%) [12]. Diaphragmatic hernia beyond the neonatal period varies from 5% to 30% [12]. In adulthood, except for CDH, there are different reasons such as trauma, phrenic nerve palsy, and delayed diagnosis of hiatus hernia for the development of diaphragmatic hernia [13]. According to the results of an extensive study on patients with CDH, a very small number of patients with ratio of 1 : 8 for men to women are diagnosed at older ages, and with regard to this study, a case with the same age as our patient is very rare [6]. X-ray image (radiography) is the most common imaging method to study diaphragm and heart. When the chest X-ray images are not diagnostic, spiral CT and MRI will be our next selections, respectively, to acquire more information about the disease. In patients with Bochdalek hernia, the diaphragm is ruptured and there is a defect in it and the small intestine moves into thoracic cavity more than any other abdominal organs [5, 14]. In order to prevent the serious complications of CDH, surgical treatments should be used. Choosing the best treatment method for repairing CDH has become a challenge among the surgeons. Some of them support thoracotomy as the best treatment option because the chest is probably attached to hernia sac [14], while some others believe that laparotomy is better than thoracotomy for dealing with possible complications such as malrotation, obstruction, strangulation, and perforation of abdominal viscera [15]. Furthermore, in some cases, surgical techniques with minimal invasion such as thoracoscopy and laparoscopy are used to repair Bochdalek hernia [16]. In the current patient, because of delayed clinical manifestation and the probability of adhesion to the chest and restoration of the diagram on the right side, we decided to perform thoracotomy to have the greatest chance of survival and minimum side effects for the patient.

## 4. Conclusion

CT and CXR were helpful for the detection of congenital diaphragmatic hernia. However, definitive diagnosis of hernia before surgery is difficult because of its infrequency and various manifestations. This issue reveals the diagnostic problem of physicians when facing the delayed CDH.

As a result, physicians should always keep this rare hernia in their minds as one of the most important recognitions. In order to improve the quality of medical cares for adult patients with congenital diaphragmatic hernia, it is recommended to follow up the patients for a long term and to report more cases.

## Figures and Tables

**Table 1:** Clinical presentation of congenital diaphragmatic hernia in adults.

	Morgagni (%)	Bochdalek (%)
Asymptomatic	28	14
Pulmonary symptoms	**36**	**37**
Pain/pressure^*∗*^	37	69
Obstruction	**20**	**39**
Dysphagia	3	3
Strangulated	**0**	**28**
Bleeding	1	4
Gastroesophageal reflux disease (GERD)	**1**	**4**
Other (HTN, fatigue, indigestion)	1	9
Symptoms for less than 1 month	28	47

^*∗*^Located in chest or abdomen, not related to obstruction/strangulation.
